# Magnetic Anisotropies and Exchange Bias of Co/CoO Multilayers with Intermediate Ultrathin Pt Layers

**DOI:** 10.3390/ma16041378

**Published:** 2023-02-07

**Authors:** Dimitrios I. Anyfantis, Camillo Ballani, Nikos Kanistras, Alexandros Barnasas, Ioannis Tsiaoussis, Georg Schmidt, Evangelos Th. Papaioannou, Panagiotis Poulopoulos

**Affiliations:** 1 Department of Materials Science, School of Natural Sciences, University of Patras, 26504 Patras, Greece; 2 Institut für Physik, Martin-Luther Universität Halle Wittenberg, Von-Danckelmann-Platz 3, 06120 Halle, Germany; 3 Department of Physics, School of Natural Sciences, Aristotle University of Thessaloniki, 54124 Thessaloniki, Greece; 4 Interdisziplinäres Zentrum für Materialwissenschaften, Nanotechnikum Weinberg, Martin-Luther University Halle-Wittenberg, 06120 Halle, Germany

**Keywords:** ultrathin films, growth, sputtering, magnetic multilayers, magnetic anisotropy, exchange bias, Co, CoO, Pt

## Abstract

Co/CoO multilayers are fabricated by means of radio-frequency magnetron sputtering. For the formation of each multilayer period, a Co layer is initially produced followed by natural oxidation. Platinum is used not only as buffer and capping layers, but also in the form of intermediate ultrathin layers to enhance perpendicular magnetic anisotropy. Three samples are compared with respect to the magnetic anisotropies and exchange bias between 4–300 K based on superconducting quantum interference device magnetometry measurements. Two of the multilayers are identical Co/CoO/Pt ones; one of them, however, is grown on a Co/Pt “magnetic substrate” to induce perpendicular magnetic anisotropy via exchange coupling through an ultrathin Pt intermediate layer. The third multilayer is of the form Co/CoO/Co/Pt. The use of a “magnetic substrate” results in the observation of loops with large remanence when the field applies perpendicular to the film plane. The CoO/Co interfaces lead to a significant exchange bias at low temperatures after field cooling. The largest exchange bias was observed in the film with double Co/CoO/Co interfaces. Consequently, significant perpendicular anisotropy coexists with large exchange bias, especially at low temperatures. Such samples can be potentially useful for applications related to spintronics and magnetic storage.

## 1. Introduction

The magnetic anisotropy of ultrathin magnetic films and multilayers prefers out-of-plane or in-plane magnetization depending on the film thickness or temperature. Because of the practical implications for high-density magneto-optical storage media, the magnetocrystalline anisotropy of ferromagnetic materials, including transition metals, has become the topic of significant theoretical and experimental research [1,2,3,4]. Potential materials for these applications must have a significant perpendicular magnetic anisotropy (PMA), which is caused by an inherent magnetic anisotropy in the crystal lattice that is strong enough to overcome the extrinsic macroscopic shape anisotropy that favors in-plane magnetization [5,6]. Such films have always been of great interest in vertical magneto-optic recording media [1,7,8,9], perpendicular magnetic tunnel junction [10,11,12], and spin-valve [13,14] devices. Significant PMA has been observed in periodically altered ferromagnetic (FM) and noble metal multilayers such as Co/Pt, CoNi/Pt, Co/Pd, and Co/Au [12,15,16,17,18,19,20]. In these multilayer systems, strong PMA is caused by an increase in the orbital moment of Co because of hybridization between the transition metal and the orbitals of heavy metals at the interface. Most PMA systems with ultrathin oxide layers have also been used in magnetic tunnel junctions [20,21].

Ferromagnetic Co/Pt multilayers and CoPt alloys present large PMA [22,23,24]. When they are in contact with an antiferromagnetic (AFM) layer such as CoO or NiO, and after field cooling through the Néel point of the AFM in an applied magnetic field, perpendicular exchange bias (PEB) is observed [25,26,27,28,29,30]. This well-known exchange bias (EB) effect is related to the magnetic coupling across the common interface shared by an FM and an AFM layer that was discovered by Meiklejohn and Bean in 1956 [31] and results in a shift (biasing) of the hysteresis loop to negative or positive direction with respect to the applied field and increased coercivity [32].

In the present work, the magnetic behavior of Co/CoO/Pt multilayers was investigated. These systems consist of ferromagnetic (FM)/Pt, antiferromagnetic (AFM)/Pt, and FM/AFM (Co/CoO) interfaces. CoO is an antiferromagnet with a Néel temperature of 270 K, close to the room temperature (RT), in bulk form [33]. The Co/CoO unit in our systems was fabricated by the natural oxidization procedure of Co at 2 × 10^−3^ mbar pressure of air kept for 1 min [34,35,36]. This method works well for both Ni and Co and results in the formation of an ultrathin oxide layer [34,35,36]. Such multilayers with Pt or thin oxide layers are stable with time and moderate annealing [37,38,39]. PMA at magnetic metal/oxide interfaces has attracted more and more scientists in recent years [40,41,42,43]. Lv et al. and Nistor et al. studied the influence of annealing on the PMA of Co/native oxide/Pt multilayers and oxide/Co/Pt thin-film structures, respectively [44,45]. Both found strong PMA enhancement caused by the Co and O atomic hybridization at the interface in ultrathin magnetic layers. On the other hand, Kumar et al. obtained significant PMA in a Co/CoO/Co thin-film structure with a thick Co film in the as-deposited state without post-annealing of the structure because the growth in Co on the oxidized interface takes place with preferential orientation of *hcp* c-axis perpendicular to the film plane [21].

In our work, ultrathin Co/CoO/Pt multilayers grown on Si (001) substrate via magnetron sputtering are found to present PMA at RT with a coercivity enhancement at a range of 4–300 K. The multilayers show considerable exchange bias at low temperatures. The origins of the enhanced PMA and exchange bias are discussed.

## 2. Materials and Methods

**Figure 1:**
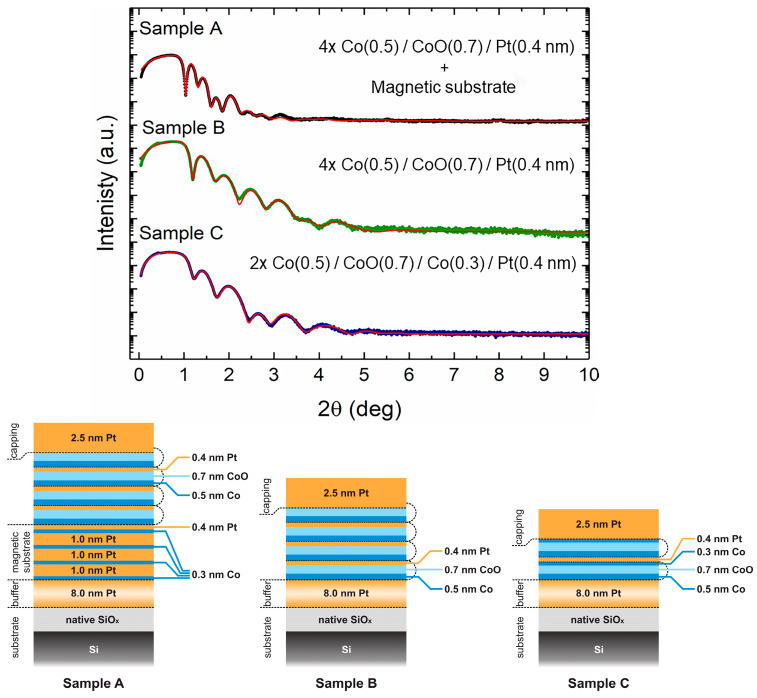
XRR patterns (up) and film drawings (down) for three Co/CoO/Pt multilayers with different formation and repetitions. The fitted patterns using the GenX code are also shown (continuous thin lines). The patterns of samples B and C have been vertically shifted for clarity of presentation.

Co/CoO/Pt multilayered samples with repetition N ranged from 2 to 4 were fabricated using a high-vacuum radio-frequency (RF) magnetron sputtering system with a base pressure of 3 × 10^−7^ mbar. Pure argon 6N gas was used for sputtering. The Ar pressure was kept constant for both metals at 1 × 10^−2^ mbar. Co and Pt deposition rates and RF power are 0.75 (30 W) and 1.5 Å/s (20 W), respectively. Film thickness was determined using a quartz balance system (Inficon XTM/2, Kurt J. Lesker Company, Jefferson Hills, PA, USA). Corning glass and Si (001) wafers with a native oxide layer were used as substrates. An 8 nm Pt buffer layer was first deposited on each substrate, followed by the deposition of Co/Pt multilayers and/or Co/CoO/Pt (Co/CoO with intermediate Pt layers). Three samples with different structures were studied in detail. The exact stackings of the three multilayers are presented schematically in Figure 1.

The periodicity and the layering quality of multilayers were determined with the aid of the X-ray reflectivity (XRR) technique. XRR measurements were performed using a Bruker D8 diffractometer (Bruker, D8-Advance, Karlsruhe, Germany) and focused Cu-Kα1 radiation (λ = 1.5418 Å).

The magnetic properties of films were measured with a Quantum Design SQUID VSM magnetometer ( Quantum Design, Darmstadt, Germany) in applied fields of up to 40 kOe in the temperature range of 4–300 K. The measurement protocol included initially cooling down in the field, setting the temperature value, and then the magnetic hysteresis loop runs from +maximum saturation field to -maximum field and back to +maximum field. In such a way, we exclude any experimental factor that could influence the magnetization reversal.

Finally, a Jeol 1010 (100 KV) transmission electron microscope (TEM) (Jeol Ltd., Tokyo, Japan) was used to investigate the structure of a selected multilayered sample in the cross-section geometry (XTEM).

## 3. Results

Figure 1 plots the XRR patterns of our multilayers deposited on the Si (001) substrate. Very clear XRR oscillations are observed for all the samples. Well-defined interfaces are needed to produce such oscillations. Since there are various individual layer thicknesses in each sample, it is not easy to separate the Bragg and Kiessig fringes [46]. In other words, there is an overlap between them. Therefore, to derive quantitative information about the total or individual layer thickness and the bilayer roughness of our samples, we performed fittings of the XRR patterns using the GenX software package [47]. The values derived from GenX fitting are the ones we provided in the previous section. These values were in fair agreement with the indications of a pre-calibrated quartz balance system for the determination of growth rates during deposition. The root-mean-square (RMS) roughness values of the Co, Pt, and CoO layers are under 1 nm (0.57–0.75 nm) for all samples.

**Figure 2:**
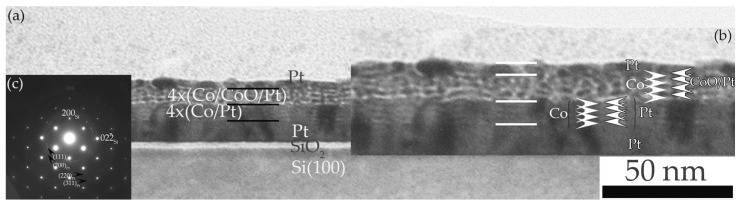
(**a**) XTEM image of sample A. One may see the Si substrate, native oxide, Pt buffer layer, “magnetic substrate”, Co/CoO/Pt multilayer, and Pt capping layer. (**b**) A magnified part of (**a**). (**c**) Electron diffraction pattern.

X-ray reflectivity works in the reciprocal space. As it is always easier to understand images, in Figure 2 we present XTEM for the most representative sample A. In Figure 2a, we observe columnar growth of Pt followed also in the multilayer. We can see the four repetitions of the “magnetic substrate”, the four repetitions of Co/CoO with intermediate Pt layers, and the Pt capping layer. Some waviness is observed because of columnar growth. Figure 2b shows a magnified part of the area. The sub-layers are indicated by arrows showing the sequence of Co/Pt first and, second, the Co/CoO/Pt close to the top. In Figure 2c, the SAED (selected area electron diffraction) pattern shows the Si substrate with a ZA = [110]. Four rings of the first (111), (200), (220), and (311) planes of the nanocrystalline platinum are visible, with the experimental measured values being 2.27 Å, 1.96 Å, 1.37 Å, and 1.16 Å, respectively.

**Figure 3:**
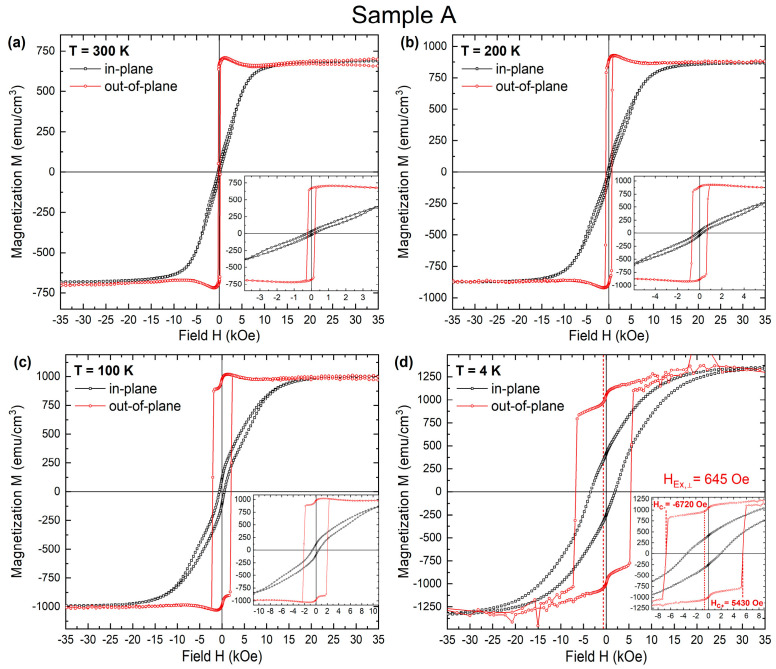
Hysteresis curves of sample A for both in-plane and out-of-plane geometries recorded by SQUID at (**a**) 300 K, (**b**) 200 K, (**c**) 100 K, and (**d**) 4 K.

Figure 3 shows the typical magnetization hysteresis loops of sample A measured at the range of 4–300 K (Figure 3a–d). The loops were recorded by SQUID with the external field perpendicular and parallel to the film plane, and after field cooling of 4 T. The magnetization easy axis lies perpendicular to the film plane, as can be seen from the square-like out-of-plane hysteresis loop. The M-H curves shown in Figure 3c,d measured at 100, 4 K after the field cooling application, exhibit enlarged perpendicular coercivity and an obvious loop shift, which is evidence for the relatively large exchange bias effect at the Co/CoO interface and significant interlayer coupling between Co/Pt and Co/CoO/Pt multilayers through the very thin Pt layer (0.4 nm) between them. At 4 K (Figure 2d), significant PEB (H_e_ ~ 650 Oe) is detected. The loop of the multilayer is rectangular with a squareness ratio of close to unity and the film has a substantial coercivity field (H_c_ ~ 6 kOe), while the in-plane hysteresis loop is sheared with a large saturation field showing hard-axis behavior. The result demonstrates that strong PMA is inherently established in the Co/CoO/Pt multilayer on top of the “magnetic substrate,” which was sputter-deposited at room temperature.

**Figure 4:**
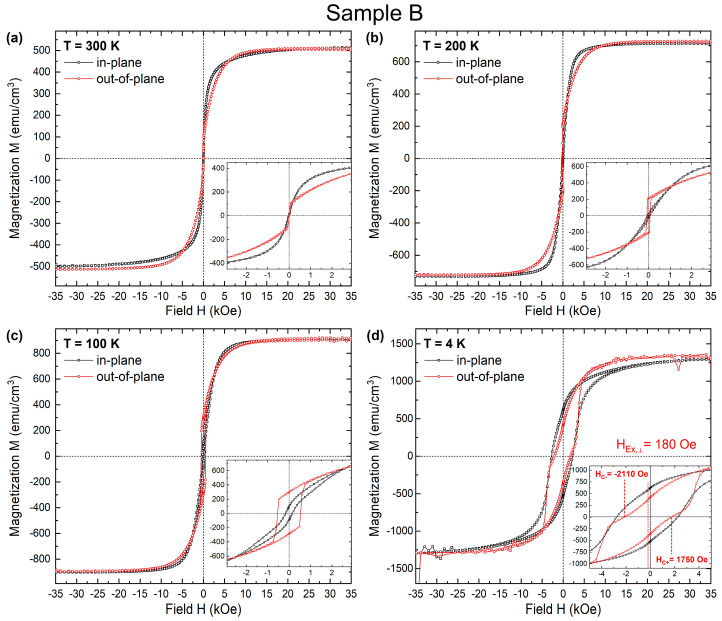
Temperature-dependent magnetization hysteresis loops with the external field applied perpendicular (red circles) and parallel (black squares) to the film plane for sample B.

In Figure 4, we plot the SQUID hysteresis loops for sample B (a) at room temperature, 300 K, (b) 200 K, (c) 100 K, and (d) 4 K. This multilayer seems to have a magnetization easy axis between in- and out-of-plane direction at RT. The perpendicular component predominates at most temperatures, with a higher out-of-plane remanence magnetization Mr than the in-plane component (Figure 4b). At 4 K, the hysteresis loops show a significant increase in coercive (H_c_) field and remanence in both applied fields parallel and perpendicular to the film plane. A weak longitudinal exchange bias (LEB) of about 230 Oe has also appeared; PEB is about 180 Oe. SQUID data reveal that sample B has an easy magnetization axis parallel to the film surface at RT. It is also important to note that sample A has higher remanent magnetization and coercive field compared to sample Β in the perpendicular direction. This is explained by the increase in Co layer thickness caused by the “magnetic substrate” of sample A that enhances the perpendicular anisotropy.

**Figure 5:**
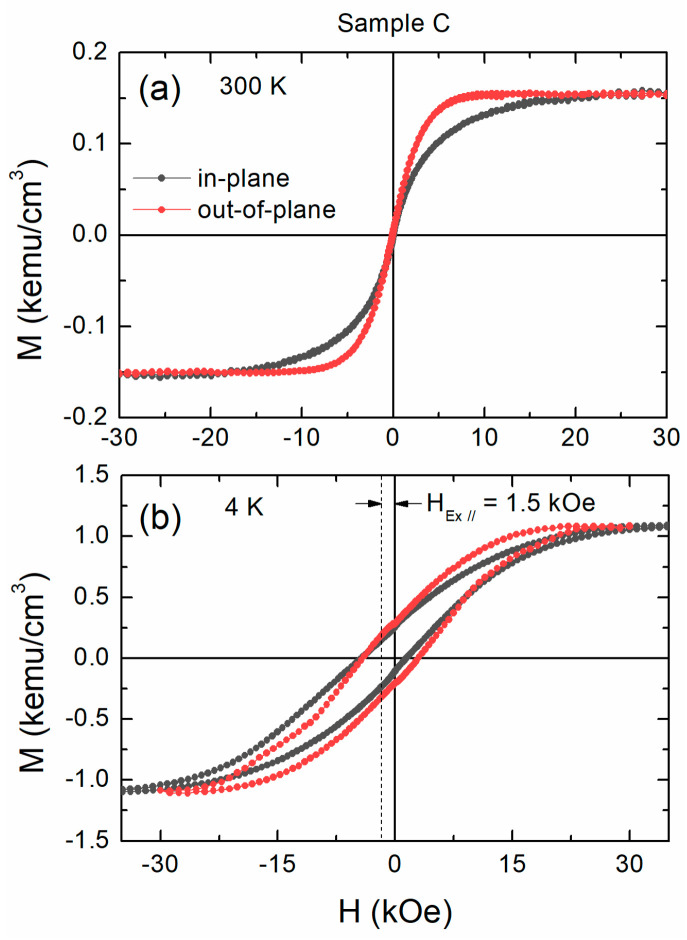
Temperature-dependent magnetization hysteresis loops with the external field applied perpendicular (red circles) and parallel (black circles) to the film plane for sample C.

In Figure 5, we plot the SQUID hysteresis loops for sample C (a) at 300 K and (b) at 4 K. This multilayer magnetizes easier along the out-of-plane direction at 4K, but magnetic remanence is much smaller than unity. No hysteresis appears at RT. Moreover, magnetization is significantly decreased at RT. This shows that RT is close to the magnetic transition temperature of the sample—that is, close to the Curie temperature T_C_. This is not surprising since some Co layers are as thin as 0.3 nm, or, just more than a single monolayer. Finite-size effect is commonly observed in ultrathin layers and is responsible for the decrease in T_C_ and the RT saturation magnetization as film thickness decreases [48].

**Figure 6:**
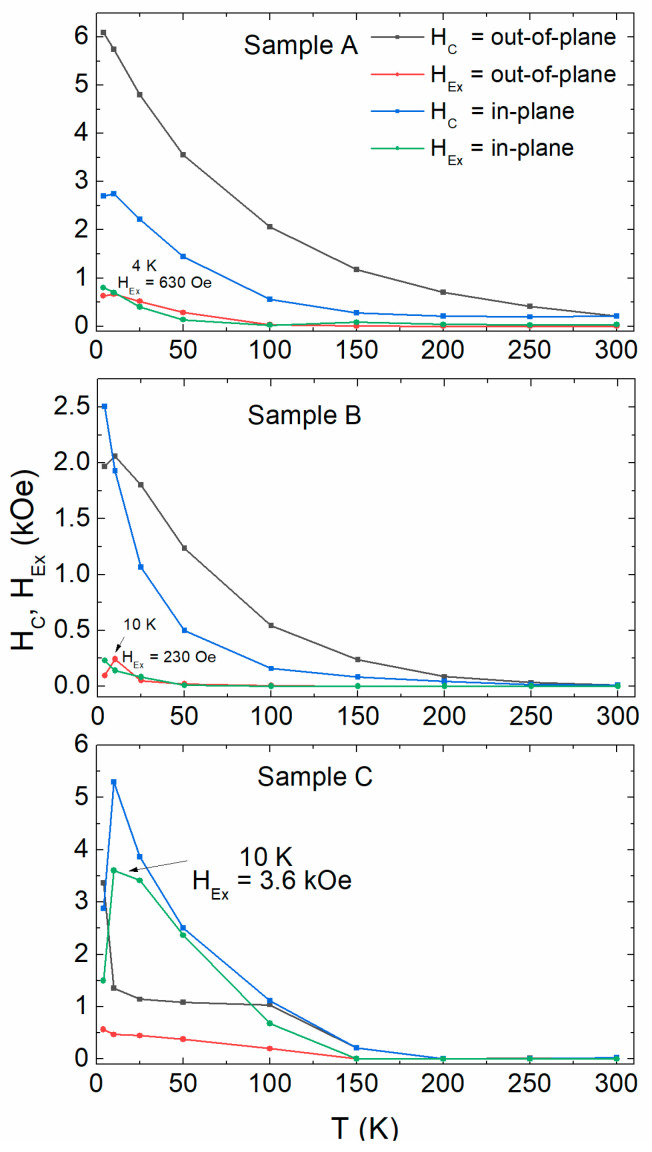
Temperature-dependent coercivity and exchange bias field for all samples measured with the external field parallel and perpendicular to the film plane. Lines serve as guides to the eye.

In Figure 6, we plot the coercivity H_c_ and exchange bias H_Ex_ fields derived from the SQUID hysteresis loops for the three multilayers between 4–300 K. At low temperatures, all samples are hard magnets. This has to do with the very thin intermediate layers of Pt. The relatively fast attenuation of the coercivity as we approach RT is due to the finite-size effect and decreased T_C_ caused by ultrathin Co layers. The double interface Co/CoO/Co in sample C seems to result in a very large exchange bias, especially in the in-plane magnetization direction.

**Figure 7:**
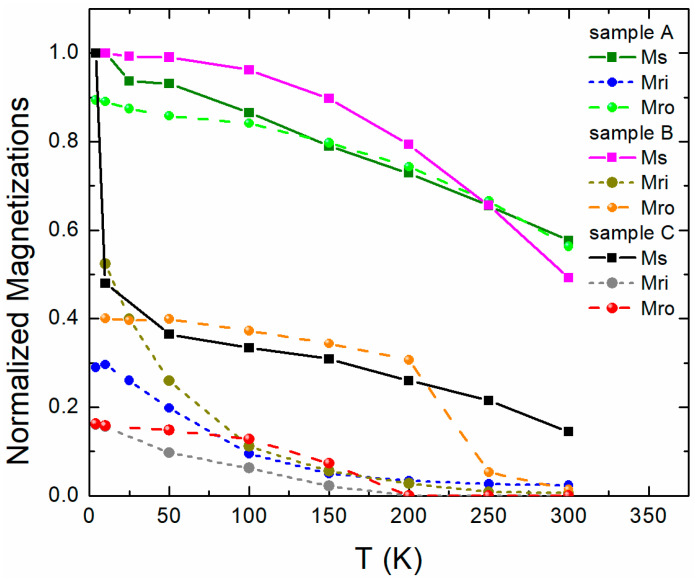
Temperature-dependent normalized saturation magnetizations Ms and remanences Mr along the in-plane (i) or out-of-plane (o) directions. Lines serve as guides to the eye.

Finally, in Figure 7, we plot the saturation magnetization Ms of the samples together with the remanence Mr values in the in- (i) and out-of-plane (o) directions to comprehend the magnetic behavior of our spin-engineered multilayers. We normalized these quantities by dividing them by the Ms of each sample at the lowest temperature measurement, so that a meaningful comparison is feasible. For sample A, the Mro is almost as large as Ms, revealing the large PMA of the sample. In sample B, Mro is larger than Mri; however, as aforementioned, this sample has an intermediate behavior. Interestingly, sample C presents a very large and unusual increase in Ms at very low temperatures. This reminds us of a similar behavior of Fe/V(001) superlattices with ultrathin Fe layers [49]. It may indirectly suggest that the thinnest layers of Co (0.3 nm) at the interface with CoO may break into islands, showing a low ordering temperature below 50 K compared to the layers of thicker Co (0.5 nm) in the same sample, which are magnetic up to room temperature (see Ref. [49]).

## 4. Discussion

Co/Pt multilayers are well-known for high coercivities, PMA, and large magneto-optic Kerr rotation, and they are mainly used in the computer hard disc storage industry (see, for example, references [1,4,15,24] among many others). On the other hand, Co/CoO multilayers with *fcc* Co stacking do not show PMA before or after mild thermal annealing [36,37]. As CoO is an antiferromagnet, exchange bias is possible to develop at the Co/CoO interface. Therefore, the engagement of the good properties of Co/Pt for PMA and large coercivity with the exchange bias of Co/CoO result in the observation of all good features for applications in the Co/CoO system with intermediate Pt layers. The use of a Co/Pt multilayer as “magnetic substrate” strongly coupled to the Co/CoO multilayer grown on top of it resulted not only in square loops in the out-of-plane magnetization direction but, also, to exchange-shifted loops.

The two-step switching for the perpendicular loop of sample A is not easy to interpret in a straightforward manner, i.e., to conclude that the two multilayers are decoupled. If one would like to make some estimate of this coupling, one has to recall that while in the magnetic substrate Pt is 1 nm thick, the last Pt layer before the starting deposition of Co/CoO/Pt is only 0.4 nm thick, i.e., two atomic layers only. Therefore, the coupling between the magnetic substrate and the top cannot be negligible. Platinum in proximity to Co for such thin Pt layers is strongly spin polarized so it has an induced moment comparable to the one of a magnetic element (see, for example, Wilhelm et al. [50]). Therefore, the coupling between the magnetic substrate and the Co/CoO/Pt multilayer is expected to be larger than a mere weak interlayer exchange coupling. Moreover, in Ref. [51], it is shown how one can measure the magnitude of coupling and an exchange shift even when the loops show two-step switching and look symmetrical. We observed a shift for the total loop. If coupling between the two multilayers was very weak, no exchange shift (attributed to Co/CoO) of the out-of-plane square loops, attributed primarily to Co/Pt multilayer, would have been observed. The two-step switching may then indicate that the magnetization of the top multilayer is a little tilted from the out-of-plane direction.

On the other hand, in samples B and C, if no “magnetic substrate” is used, we still have enhanced PMA, though without square hysteresis loops, solely, this time, because of the intermediate Pt layers. By increasing the number of Co/CoO interfaces, as in sample C, the exchange shift is enhanced. The coercivity increase in sample C as compared to sample B may have to do with more increased frustration because of the double Co/CoO interface [52]. At this point, an interesting question could be why this large H_Ex_ has been observed at low temperature but fades out at RT. We think that a finite-size effect is one possible explanation. As shown by Alders et al. [53], five monolayers of NiO have a reduction in the Néel point T_N_ to about T_N_ /2 as compared to bulk. Since we have similarly thin CoO layers, their T_N_ should be then about 150 K. In this sense, maybe one must increase the thickness of the oxide layer. For example, Belhi [54] managed to obtain exchange bias at RT by using a 4 nm thick NiO layer. A second possible explanation could be the decrease in blocking temperature T_B_ with the use of very thin CoO layers as, for example, Zhang et al. have shown [55]. The increase in both T_N_ and T_B_ by increasing the NiO thickness have been discussed in Ref. [56] and references therein. As a perspective of the present work, future investigation could then have to deal with the use of thicker CoO or NiO layers.

## 5. Conclusions

The magnetic anisotropies and exchange bias of Co/CoO naturally oxidized multilayers with intermediate ultrathin Pt layers were studied by means of temperature-dependent SQUID magnetometry between 4 and 300 K. Samples were produced by radio-frequency magnetron sputtering in high vacuum. Three variations of multilayers were fabricated. It was shown that the use of intermediate Pt layers resulted not only in the enhancement of perpendicular magnetic anisotropy and coercivity of Co/CoO multilayers at all temperatures, but also in a significant exchange bias at low temperatures. Such samples can be potentially useful for applications related to spintronics and magnetic storage.

## Data Availability

Data sharing not applicable.
